# Supplementary material for: Identification and functional analysis of non-coding regulatory small RNA FenSr3 in Bacillus amyloliquefaciens LPB-18
Source: PeerJ. 2023 May 15;11:e15236. doi: 10.7717/peerj.15236 (PMC10194069; doi:10.7717/peerj.15236)
Supplement: Supplemental Information 4 [file peerj-11-15236-s004.zip › KO/CK-vs-T1_map/map02025.html]

KEGG PATHWAY: Biofilm formation - Pseudomonas aeruginosa - Reference pathway


|  |  |
| --- | --- |
| **Biofilm formation - Pseudomonas aeruginosa - Reference pathway** |  |

[
Pathway menu
| Organism menu
| Pathway entry
| Show description
| User data mapping
]

Surface colonization and subsequent biofilm formation and development provide numerous advantages to microorganisms. The main key actors relevant in the regulation of biofilm formation by Pseudomonas aeruginosa include cAMP/Vfr signaling, quorum sensing (QS) systems, Gac/Rsm pathway, and c-di-GMP signaling. cAMP/Vfr signaling regulates the transcription of hundreds of genes encoding diverse virulence factors, including the type 2 secretion system and type 3 secretion system and their associated toxins, type IV pili, and flagella. It is demonstrated that the accumulation of cAMP inhibits the attachment phase of biofilm formation. The QS systems are arranged hierarchically with the las system positively regulating both the rhl and PQS systems. These three QS systems are involved in the regulation of virulence factor production, biofilm maturation, and motility phenotypes. GacS/GacA two-component system is promoting the expression of two small regulatory RNAs, RsmY and RsmZ, which sequester the translational repressor RsmA. Titration of RsmA induces the production of sessile and biofilm determinants, whereas free RsmA leads to a planktonic and more virulent lifestyle. c-di-GMP is a ubiquitous bacterial second messenger. Synthesis and degradation of c-di-GMP is facilitated by diguanylate cyclases (DGC) and phosphodiesterases (PDE). High intracellular levels of c-di-GMP evoke the processes leading to the production of biofilm matrix, while a decrease in its level causes an increase in cell motility and transition into the planktonic forms of growth.

|  |  |  |
| --- | --- | --- |
| Reference pathway | 184% 150% 122% 100% 82% 67% 55% | 图片下载 |
